# Integrating smartwatch-based out-of-hospital cardiac arrest detection into resuscitation systems: a focus group study of community responder perspectives

**DOI:** 10.1016/j.resplu.2025.101027

**Published:** 2025-07-08

**Authors:** Marijn Eversdijk, Babette J.W. van der Eerden, Dick L. Willems, Willem J. Kop, Mirela Habibović, Marieke A.R. Bak

**Affiliations:** a Department of Medical and Clinical Psychology, Center of Research on Psychological Disorders and Somatic Diseases, Tilburg University, Tilburg, the Netherlands; b Department of Ethics, Law and Humanities, Amsterdam UMC, University of Amsterdam, Amsterdam, the Netherlands; c Institute of History and Ethics in Medicine, Department of Preclinical Medicine, TUM School of Medicine and Health, Technical University of Munich, Munich, Germany

**Keywords:** Out-of-hospital cardiac arrest, Community responders, Medical ethics, Digital health, Smartwatches

## Abstract

**Background:**

Smartwatch-based solutions for out-of-hospital cardiac arrest (OHCA) detection are increasingly explored to enhance survival outcomes, especially in unwitnessed cases. However, the perspectives of community responders, who play a critical role in early responding to resuscitation alerts, are not yet known. Exploring their current experiences and perspectives on this new technology is important for successfully developing and integrating these innovations into existing resuscitation systems.

**Methods:**

Four semi-structured focus group discussions (FGDs) were conducted with 15 community responders in the Netherlands, recruited through the national cardiac arrest emergency network. Each FGD comprised two discussion topics: (1) integrating smartwatch-based OHCA detection into the current resuscitation system and (2) mitigating the burden of this technology on community responders. Verbatim transcripts were inductively coded for recurring themes.

**Results:**

The FGDs revealed 54 unique codes that were grouped into four themes: (1) Missing information about the cause of the alert; (2) decision-making regarding accepting the alert, (3) challenges in reaching the location and (4) availability of aftercare for community responders.

**Conclusion:**

This study identifies target topics that require attention in order to optimise community responders involvement in the implementation of smartwatch-based OHCA detection. Community responders indicated communication with the emergency dispatch centre, training with smartwatch-initiated scenarios and opt-in functionalities for responding to potential impactful situations as key factors to enhance the acceptability and feasibility. These factors ultimately aim to facilitate faster and more effective resuscitation following smartwatch-initiated alerts.

## Introduction

Out-of-hospital cardiac arrest (OHCA) is a major health problem, with an incidence of approximately 40–100 individuals per 100,000 each year in the general population.^1^, ^2^, ^3^ Survival chances are estimated between 4.6 % and 16.4 %, but there is a large variation between European countries.^2^ In the Netherlands, survival chances have increased towards 23–27 %, as a result of bystander resuscitation, automated external defibrillator (AED) use at the scene and technological innovations.^4^ Enhanced bystander resuscitation can be achieved through the implementation of a first responder dispatch system, which has been associated with increased rates of return of spontaneous circulation and survival following hospital discharge.^5^ Wearable-based solutions for the early detection and start of the chain of survival after OHCA are now becoming available for implementation, and several research groups are currently investigating effectiveness and feasibility-related issues.^6^, ^7^, ^8^

To achieve the fastest dispatching of responders on site in unwitnessed events, it could be beneficial to incorporate smartwatch-based or other wearable solutions into current resuscitation alert systems.^7^, ^8^, ^9^ This would involve first responders of both regular emergency dispatch systems, referred to as on-duty responders (e.g., ambulance, police, firefighters), as well as first responders in volunteer-based systems (e.g., HartslagNu), referred to as community responders.^10^ Our previous literature review on the integration of smartwatch-based OHCA detection has highlighted that issues related to device accuracy and false alarms, psychological wellbeing of the wearer, privacy and consent, and equitable access need to be solved to ensure the successful integration into resuscitation systems.^11^ These challenges are anticipated to impact various stakeholders across the chain of survival, necessitating coordinated efforts to address their implications for resuscitation practise and technology development.

Despite their critical role in the early initiation of basic life support (BLS),^5^ community responders have not yet been optimally involved in discussions on integrating smartwatch-based OHCA detection into resuscitation systems. This is problematic as their frontline experiences offer valuable insights for improving both current practises and the successful adoption of emerging technologies.^12^ Moreover, integrating smartwatch-based OHCA detection may introduce new challenges for responders, including false alarms, restricted access due to locked doors, and being solely responsible for providing basic life support in the absence of other bystanders. Furthermore, as community responders engage in resuscitation efforts voluntarily, it is essential to consider the psychological impact of their involvement and the factors that influence their continued motivation to respond to alerts. Exploring prior experiences of community responders and their views on integrating smartwatch-based OHCA detection would give valuable insights for solving these challenges in the development of the technology.

The current study therefore investigates the perspectives of community responders on wearable technologies aimed at automatically detecting OHCA and alerting the chain of survival. The aim of the study is to provide themes that are important to consider in recommendations for integrating smartwatch-based alerts into resuscitation systems and further increase chances of survival after OHCA.

## Methods

### Design

**Fig. 1:**
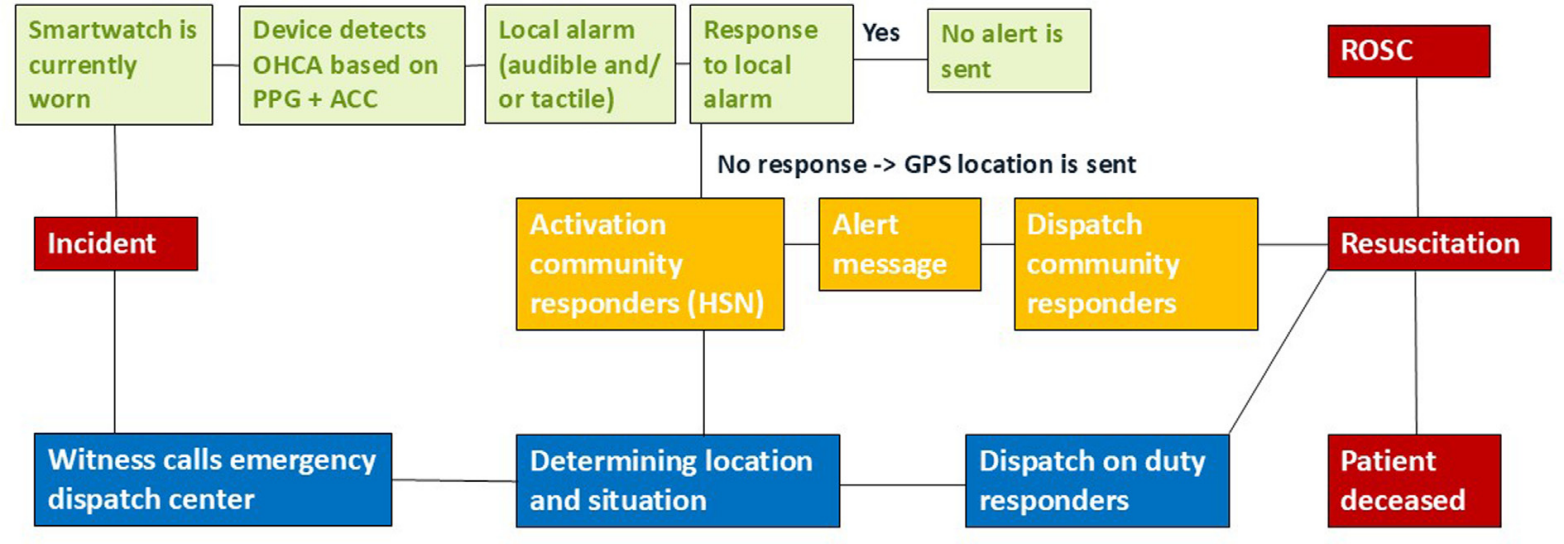
Visualisation of how the BECA platform aims to integrate smartwatch-based OHCA detection into the chain of survival, with the dispatch system of on-duty responders in blue, community responders in yellow and smartwatch-based initiation in green. Abbreviations: PPG, photoplethysmography; ACC, accelerometery; GPS, global positioning system; HSN, the network of OHCA response system partners HartslagNu; ROSC, return of spontaneous circulation. (For interpretation of the references to colour in this figure legend, the reader is referred to the web version of this article.)

Qualitative focus groups on community responder perspectives were conducted as part of the BEating Cardiac Arrest (BECA) project. The BECA project, in which all authors are involved, is a consortium that aims to develop smartwatch technology to detect a cardiac arrest autonomously and subsequently alert emergency dispatch services (Fig. 1).^7^

Four focus group discussions (FGDs) were conducted between October 2024 and March 2025, with 15 community responders in total. The consolidated criteria for reporting qualitative research (COREQ) were followed to ensure transparency and rigour of study reporting.^13^

### Participants and procedure

Participants were enrolled through *HartslagNu* [EN: HeartbeatNow], a resuscitation alert system for dispatching community responders.^14^ Participants were approached by e-mail by a project leader at HartslagNu based on the following inclusion criteria: proficiency in Dutch, age above 18, at least one previous experience with resuscitation from a call initiated by HartslagNu and being an active responder within proximity (maximum of 40 km) of the cities where the FGDs took place (Amsterdam, Tilburg, Rotterdam, Nijmegen; chosen based on variation in geographical position in the Netherlands and population density). There were no a priori sampling criteria related to distributions in age, gender or experience level within each FGD. Data saturation was assessed after each FGD session to decide whether an additional FGD was needed, which ended after four FGDs due to the large homogeneity within the sample.

The invitation e-mail already included detailed study information and informed consent forms. After responding, participants were contacted through a phone call with an involved researcher (BvE) to discuss participation, practical matters and current experiences as a community responder, to estimate eligibility and to provide participants the opportunity to ask questions. The FGD was scheduled when a minimum of six participants had expressed willingness to participate. For two FGDs, achieving this minimum required a second outreach. Written consent was obtained after participants were allowed to ask questions at the beginning of each FGD. After the FGDs, a Qualtrics link with a short questionnaire was sent by e-mail to inquire about demographic measures (i.e., age, gender, years active within HartslagNu and average resuscitations per year). To ensure confidentiality, all potentially identifiable information (e.g., names, locations) was deleted from the verbatim transcripts. The study was assessed by the Tilburg School of Social and Behavioural Sciences Ethical Review Board (protocol RP1687).

### Focus groups

All FGDs took place in person in a meeting room at a university medical center or university campus. The FGDs consisted of three to a maximum of six participants to optimise conversational interaction between participants. Our topic guide for the FGDs (Appendix A) was based on previous research on the ethical and psychological aspects of smartwatch-based OHCA detection^11^ and multiple feedback rounds with various BECA consortia members, including a developer of the HartslagNu system.

The FGD started with a short presentation on the rationale behind integrating smartwatch-based OHCA detection into the HartslagNu system and the opportunity for asking clarifying questions. In the first discussion round, participants were instructed to write anticipated challenges with the technology on a post-it and place these on a visualisation of the BECA platform (similar to Fig. 1). A group discussion followed, where participants could react to the addressed topics. The second phase started with a summary of earlier identified challenges to provide more depth to the following discussion, which consisted of a group discussion exploring various factors influencing community responders’ willingness to engage in resuscitation efforts, including motivations for initial enrolment, the psychological impact of prior resuscitation experiences, and the anticipated effects of smartwatch-based OHCA detection on responder motivation. The FGDs lasted two hours in total, with each round lasting 30–45 min. The discussion was recorded and transcribed for analysis. A summary of the interview was written and sent to the participants for input as a member check by e-mail, on which the participants had no additional comments.

### Researcher reflexivity and data analysis

All FGDs were led by the lead author (ME), a male 29-year-old PhD candidate studying the ethical and psychological aspects of smartwatch-based OHCA detection, with a background in psychology. There was no relationship established before the study between the lead author and participants. Some participants reported having seen each other before at resuscitation training. Participants knew beforehand that the FGDs would be part of a larger set of studies on the feasibility of smartwatch-based OHCA detection (BECA project).^7^ In order to reduce potential bias from identified themes in earlier research, the objective of the first round using post-its was to independently generate novel challenges. Challenges identified in earlier work within this project were shared with participants after their first brainstorming session to provide more context for the discussion in the second round. However, these topics could have affected their answers in round 2. Participants could also see that the interviewer was wearing a smartwatch, which could have affected their answers. The second author (BvE) attended the FGDs as well, by taking notes and keeping track of time.

Verbatim transcripts of the interviews were inductively coded and analysed in Atlas.ti by two researchers (BvE and ME), using a thematic analysis approach.^15^ Findings were interpreted from a pragmatic epistemology, focusing on the practical meaning and utility of themes for implementation.^16^ Both researchers kept track of a codebook, where alterations and new codes were logged. After the two researchers had independently coded the four FGDs, the researchers compared their codes to reach consensus on a definitive set of codes. After the coding phase, all codes were analysed and grouped by three researchers (BvE, MB and ME) on recurring themes for analysis (Appendix B). All three researchers had different theoretical backgrounds, including expertise in qualitative research.

## Results

### Participant characteristics

The 15 community responders ranged in age from 22 to 71 years (*M* = 51.07, SD = 12.66), with three female participants (see Table 1). Participants varied in resuscitation experience, ranging from highly experienced responders, some of whom also served as BLS instructors, to those with limited exposure, responding to alerts only a few times annually. The sample was characterised by a relatively high proportion of experienced community responders.

**Table 1:** Participant characteristics.

	Community first responders (*N* = 15)
Age (years)	51.07 (12.66)
Gender (female)	3
Education level	
High	6
Middle	8
Low	0
Number of years as an active community responder	7.13 (5.30)
Total number of resuscitations per year	4.50 (3.06)

### Identified themes

A total of 54 unique codes were identified, which were converted into four overarching themes: (1) Missing information about the cause of the alert, (2) Decision-making regarding accepting the alert, (3) Challenges in reaching the location and (4) Availability of aftercare for community responders.

#### Theme 1: Missing information about the cause of the alert

Community responders expected that the integration of smartwatch-based OHCA detection could result in a higher number of alerts with no actual OHCA (e.g., false positives). These could be either situations of unintended triggering of the alarm (e.g., pinched arm during sleep) or situations of physical trauma resulting in a loss of both pulse and movement (e.g., fatal car accident). Despite the potential for such uncertainties, participants expressed a continued willingness to respond to alerts, emphasising their commitment to providing assistance whenever needed.

*“It wouldn’t matter to me whether such a resuscitation call comes from the emergency centre or from a phone or watch. You don’t know what to expect anyway, so you just go out and check.”* (Focus group 4)

Still, community responders indicated that it could prepare them for the upcoming situation if they are aware that the alert comes from a smartwatch instead of the emergency centre. The main reason is that a smartwatch-initiated alert increases the probability of encountering unknown situations or barriers (e.g., locked doors, dogs present at the site, trouble in locating the victim), which requires a different mindset.

*“How is it communicated to us, right? […] of course, it’s nice if there’s a message saying, like, ‘Hey, it’s coming from a watch.’ … Like [other community responder] just said, you can also approach it with a slightly different mindset; it could be that you can’t get in, and you have to be able to deal with that as well.”* (Focus group 4)

Witness-initiated alerts are currently assessed and filtered by the emergency dispatch centres based on the information provided by the witness, to determine the feasibility of resuscitation by community responders (e.g. excluding cases involving children or other causes than a cardiac arrest). This system prevents community responders from arriving in dangerous or psychologically impactful situations. However, due to missing contextual information, this will not be possible for smartwatch-initiated alerts. Community responders indicated that not all of their fellow responders might be willing to or know how to deal with these calls. A solution raised to deal with these unknown causes of triggering the alarm was to make responding to these calls an opt-in functionality within the community responder app. A similar functionality is already in place for receiving alerts during sleep hours. In addition, community responders proposed to make a smartwatch-initiated scenario part of educating responders, to better prepare them for these sorts of situations.

*“In terms of training, as a community responder, you need to train with an additional protocol [on smartwatch-initiated alerts] around it, to know how to deal with the situations. I think, including locked doors and such into a well-developed protocol. So that you can at least rely on something and don’t have to think about it anymore.”* (Focus group 1)

#### Theme 2: Decision-making regarding accepting the alert

In the choice of responding to current alerts, many community responders reported that they often make a personal estimation of whether they will arrive at the scene before on-duty responders. If it would take significantly more time, they sometimes choose not to respond to a resuscitation alert. Community responders based their estimation on previous experiences, the location of the event, and whether they had already heard the sound of an ambulance nearby.

*“Yes, about those previous experiences, I’ve had it happen a few times that I arrive while the ambulance is already there or something along those lines. That has made me a bit more critical about which alerts I do respond to and which I don’t… I really make the assessment of whether I will get there faster than the ambulance.”* (on the decision to respond to a resuscitation alert and how this could change as a result of smartwatch-initiated alerts) (Focus group 3)

Community responders primarily saw their value in being the first to arrive at a site to start basic life support. After the arrival of on-duty responders, they were often instructed to hand over resuscitation to these professional on-duty responders. Observing from a distance that a sufficient number of responders are already present at the scene may lead some community responders to disengage and return home. However, participants also emphasised that an abundance of responders is not necessarily problematic, as additional responders can contribute in alternative supportive roles.

*“I’ve been the first to arrive and alone a few times already. You know, you just get to work, that’s it. You’re there for the victim, and whoever arrives next can take care of the relatives or keep people at a distance.”* (on the possibility of being the first to arrive more often in the case of smartwatch-initiated alerts) (Focus group 3)

Despite these considerations, community responders indicated that they would rather respond too many times instead of too few. They reported that whether an increase in false positives could result in less motivation to respond to subsequent alerts was difficult to answer at the current stage of technological development, as they also indicated the need to experience this first. Community responders reported that their primary aim is to help people and that they want to keep doing that. They intend to keep responding in the same way after false alarms, but reported that if a false alarm comes multiple times from the same watch, it would be wise to have this checked.

*“It’s about a human life after all. And sometimes you might respond for nothing, but how great is it that you can go and maybe help? … I don’t know if I would still rush over after 20 false alarms, but for now, you just keep responding.”* (on whether an increase in false alarms as a result of smartwatch-initiated alerts would affect motivation to respond) (Focus group 4)

#### Theme 3: Challenges in reaching the location

Community responders reported that they sometimes experience difficulties in reaching the location of the incident or collecting an AED if instructed to. The main reason is that the current system sends alerts to community responders within a certain radius, not accounting for physical barriers such as highways, train tracks or waters in between. Even after arriving at the site, it is not always easy to directly locate the victim, which is expected to be even harder when there is only a GPS location available and no address, in the case of smartwatch-initiated alerts. Providing home address information as part of the alert from the smartwatch was mentioned by multiple community responders as a complementary solution to deal with the error margins of GPS signals, in particular for buildings with multiple floors. However, it was also said that the victim might not always be at home. In those cases, providing the home address could also result in a delay of locating the victim.

*“I have sometimes not been able to find a door. That’s really annoying. I was there first. Then the police arrived, they were the second party, and they couldn’t find it either. Then we started walking around, and the firefighters came, and they were able to find it. That’s very frustrating.”* (on problems with localisation which could increase when there is only a GPS location available during smartwatch-initiated alerts) (Focus group 1)

Community responders expected a higher likelihood of stressful situations when there are no witnesses around during a smartwatch-initiated alert. Situations such as locating the victim, encountering locked doors or being the first to enter someone’s home while also taking into account personal safety could be potentially stressful endeavours. Responders indicated the need to be prepared for these sorts of situations, also keeping their own safety in mind. Potential aid in these situations could come from having contact with the emergency dispatch centre. Responders indicated that they were willing to function as a first witness on-site by providing the emergency dispatch centre with the necessary information, in return for directions on how to act.

*“Most people [community responders] just wait for instructions. And what you want in such a setting is for someone to step up and take the lead, and that’s often the issue. People do feel equipped to follow instructions … but they feel uncertain about their own actions when they are on their own…”*

*“That’s why I would also find it useful that if you are called up and you are in the middle of nowhere [which could occur more often in unwitnessed smartwatch-initiated alers], you can put the app on speaker and get connected to the emergency center, for example.”* (Focus group 1)

#### Theme 4: Availability of aftercare for community responders

Community responders all highlighted the importance of good aftercare after psychologically impactful resuscitations. Although they acknowledged people might respond differently to various impactful situations, some situations can generally be experienced as more impactful than others (e.g., involvement of children or severe physical trauma), which might require additional courses of action to keep community responders mentally healthy and willing to respond to new resuscitation alerts.

*“Aftercare is very important anyway… Everything happens very quickly, of course. And those professionals have to move on to the next thing very quickly after such a situation, because for them it’s just work. But then as a community responder you are left standing like ‘Oh, okay, I’m on my own now, what happens next? What happens to the victim?’ Some people do feel the need for aftercare, while others don’t. It’s really diverse.”* (on the potential of increased encounters of psychologically impactful events as a result of smartwatch-initiated alerts) (Focus group 1)

The use of smartwatch-based OHCA detection has the potential to increase the number of both impactful situations (as a result of no filtering by the emergency dispatch centre) and experiencing situations with worse clinical outcomes (as a result of other causes for triggering the alarm). For this reason, community responders indicated that aftercare should be given increased attention when the alert has been initiated by a smartwatch. Together with the earlier-mentioned opt-in functionality for receiving smartwatch-initiated alerts, this could ensure keeping community responders motivated and willing to respond to new alerts.

*“You also have to realise that it can have a huge impact if you see that 3 or 4 people in a row just don’t make it, which, statistically speaking, is more than likely. That also has an impact on you, and then it comes back to readiness or willingness.”* (on experiencing increased fatal outcomes as a result of smartwatch-initiated alerts) (Focus group 3)

## Discussion

**Fig. 2:**
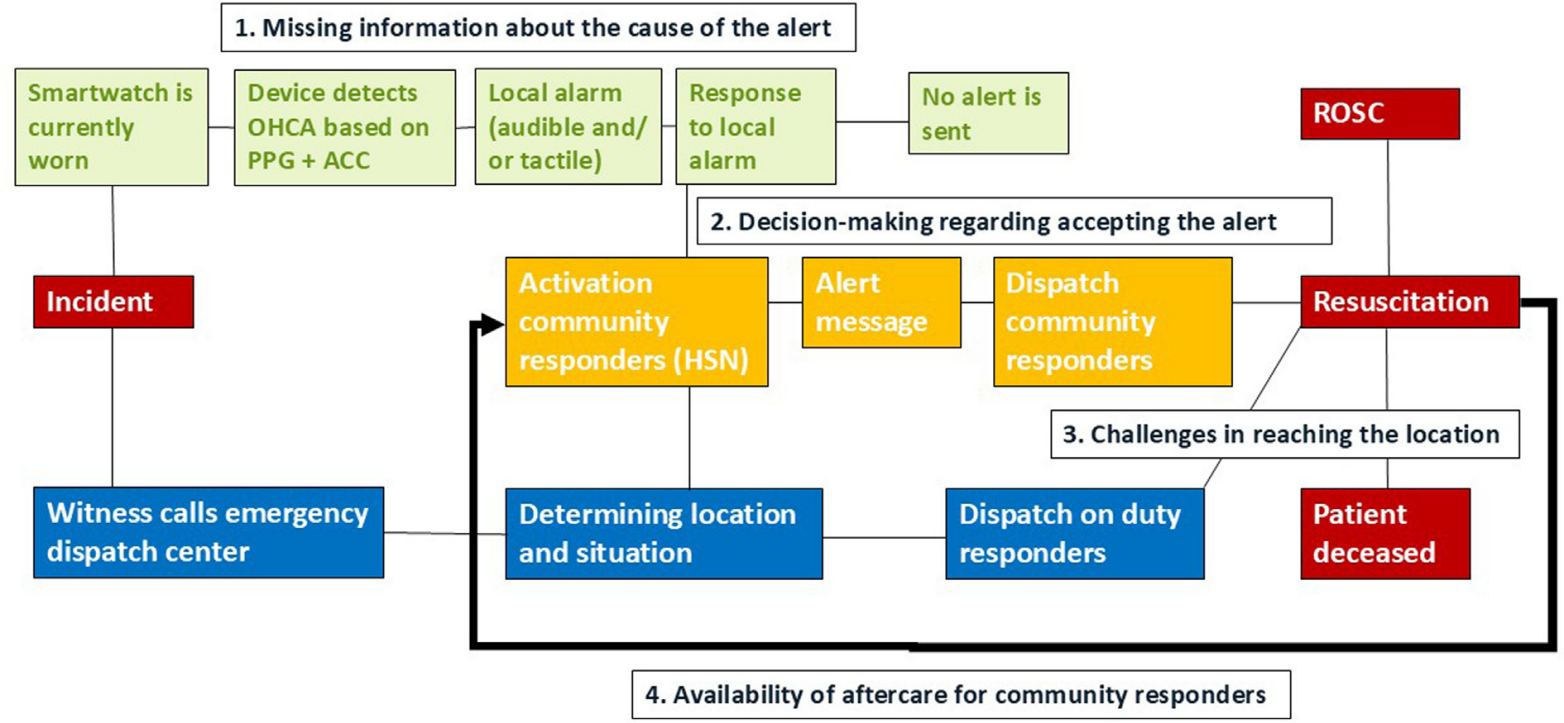
Integration of the identified themes into the BECA platform.

This study revealed four themes related to barriers and opportunities expressed by community responders when considering the integration of smartwatch-based OHCA detection. First, smartwatch-initiated alerts were expected to increase uncertainty regarding the cause of the alert (e.g., potential false positives or arriving at impactful situations). Communication with the emergency dispatch centre, training with smartwatch-initiated scenarios, and opt-in functionalities for responding to these alerts were given as potential solutions to prepare community responders for these new situations. Secondly, community responders indicated that their current decision-making in responding to alerts is based on their ability to reach the location on time. However, it remains uncertain at this stage of development whether the decision-making of responding would be influenced by smartwatch-initiated alerts. Third, it was mentioned that reaching the location of the victim could become more difficult in the case of a smartwatch-initiated alert. Lastly, community responders highlighted the need for good aftercare in the case of psychologically impactful resuscitations, especially if smartwatch-based alerts could increase the occurrence of adverse or unpredictable situations. These themes are important for the further development and implementation of automated OHCA detection using smartwatches or other wearable devices and are visualised in Fig. 2.

Existing challenges related to voluntary responding to smartphone resuscitation alerts^12^ were noted by our respondents and believed to be exacerbated when smartwatch-based alerts are introduced. Additionally, similar challenges were identified with an earlier literature review on challenges with implementing smartwatch-based OHCA detection, particularly with respect to device accuracy and the potential impact of false alarms on various stakeholders.^11^ The feasibility of integrating smartwatch-initiated alerts into first responder systems will largely depend on the number of false alarms observed during validation studies. While first studies report encouraging outcomes related to false alarms,^6^, ^17^, ^18^ the influence of false alarms on responder behaviour remains uncertain, and community responders reported uncertainty in the FGDs about how false alarms might influence their willingness to respond. Further exploration of the interaction between the technology and its users after implementation is needed, as technological changes could also lead to unintended consequences.^19^, ^20^ For example, seemingly helpful solutions such as disclosing that an alert originated from a smartwatch could also unintentionally reduce responses if perceived as a signal with a high likelihood of being a false positive.

The current focus group results also show that even in the case of a nearly perfect algorithm, there are still some practical considerations to increase acceptability and motivation among community responders. Resuscitation attempts are stressful events and could become even more stressful if hindered by troubles in localisation or being the first to arrive at a site.^21^, ^22^ Although earlier studies have reported a low prevalence of post-traumatic stress disorder (PTSD) and stress-related symptoms among community responders,^23^ ongoing monitoring of their psychological well-being remains essential, particularly after integrating smartwatch-initiated alerts into resuscitation systems.

This study has several strengths and limitations. Because of the sampling procedure, where participants had to schedule free time during working hours to participate, there was a high prevalence of expert and highly motivated community responders. Their information was highly valuable, and they provided a lot of expert knowledge, but might not be representative of all community responders within HartslagNu. There was also an underrepresentation of female community responders, whose prevalence is generally lower within HartslagNu and might have a generally lower willingness to engage in smartwatch-based OHCA detection.^24^ Although our results related to responding to smartwatch-initiated alerts did not show clear gender differences, more inclusive quota sampling procedures or alternative qualitative approaches could be a solution in future research. In addition, a short questionnaire study could also assess the general opinion towards smartwatch-based OHCA detection within the overall sample of community responders. During the FGDs, there was a high focus on current resuscitation experiences, and some participants reported finding it difficult to hypothesise how they would respond to smartwatch- or other automatically initiated calls. At the same time, community responders reported that they valued being involved in an early stage of development, as it enabled them to contribute input and help guide the development process toward solutions that align with their specific needs and circumstances.

## Conclusion

The current focus group study with community responders provided valuable insights for the implementation of smartwatch-based OHCA detection into resuscitation infrastructures. Themes spanned the entire continuum from incident detection to resuscitation, encompassing alert initiation, accepting alerts, arrival at the scene, and facilitating aftercare. Communication with the emergency dispatch centre, education with smartwatch-initiated training scenarios and opt-in functionalities with regard to acting on smartwatch-initiated alerts were given as solutions to increase the acceptability and feasibility of smartwatch-based OHCA detection among community responders, in order to achieve better and faster resuscitation following a smartwatch-initiated (unwitnessed) OHCA alert.

## CRediT authorship contribution statement

**Marijn Eversdijk:** Writing – review & editing, Writing – original draft, Visualization, Methodology, Investigation, Formal analysis, Conceptualization. **Babette J.W. van der Eerden:** Writing – review & editing, Writing – original draft, Visualization, Methodology, Investigation, Formal analysis, Conceptualization. **Dick L. Willems:** Writing – review & editing, Supervision, Methodology, Conceptualization. **Willem J. Kop:** Writing – review & editing, Supervision, Methodology, Conceptualization. **Mirela Habibović:** Writing – review & editing, Supervision, Methodology, Conceptualization. **Marieke A.R. Bak:** Writing – review & editing, Supervision, Methodology, Formal analysis, Conceptualization.

## Funding

This research project is financed by the PPP Allowance made available by Top Sector Life Sciences & Health to the Dutch Heart Foundation to stimulate public–private partnerships, grant number 01-003-2021-B005.

## Declaration of competing interest

The authors declare that they have no known competing financial interests or personal relationships that could have appeared to influence the work reported in this paper.

## Figures and Tables

**Table:** 

Round	Example text or purpose
Introduction (0–10 min)	“Good morning everyone. First of all, I would like to welcome you to this focus group discussion. We thank you for coming. My name is Marijn Eversdijk; I am a PhD candidate at Tilburg University specialising in wearable technological applications within cardiology. I will be leading this focus group, which will specifically address the application of smartwatches within the HartslagNu network, but more on that later. My colleague next to me, Babette van der Eerden, will assist with this session, take notes and keep track of the time. The focus group will last approximately 2 h and we will have our discussion today in two rounds. The first round will discuss how smartwatch technology will be integrated into the HartslagNu network and what the expected consequences of this integration are. The second round will focus on the impact of responding to an alert and how this may change as a consequence of the technology. The purpose of the focus group is to gain a clear understanding of your opinions regarding HartslagNu and the potential addition of smartwatch-based OHCA detection within this network. I would like to emphasise that there are no right or wrong answers here and I would also ask you to respect differing opinions. As the facilitator, I will try to remain neutral and give each of you the opportunity to speak as much as possible. Everything discussed here will remain confidential, and your responses will be anonymized in the final research report to ensure that they cannot be traced back to you. To ensure that no valuable information is lost, the audio of this session will be recorded. This data will be converted into a text file that is accessible only to the researchers involved. Once the text file has been processed, the audio recording will be deleted. Now that you know our names and are aware of the procedure, we would also like to know your first names. Additionally, we are curious about the amount of experience you have as a volunteer responder with HartslagNu.”
Round 1: presentation, brainstorm on pros and cons of the technology, group discussion to formulate design recommendations (30–45 min)	Purpose: In this phase, we aim to explore how smartwatches with cardiac arrest detection can be integrated into the current first responder infrastructure within HartslagNu. The session will begin with a brief presentation on the topic, concluding with a schematic visualisation of the resuscitation process within HartslagNu. Participants will be asked to brainstorm individually or in pairs about the advantages and disadvantages of the technology and write them down on different post-its. These post-its will then be placed on their own copy of the schematic to identify at which stage these aspects occur. Participants will also be asked to indicate which points they consider most important personally. A group discussion will follow to reflect on potential solutions. Aspects related to the impact of responding to an alert may be mentioned, but participants will also be asked to delve deeper into this during Round 2.

Break (0–15 min)	

Round 2: group discussion on psychological impact of responding to an OHCA alert and how the smartwatch technology could have an effect on this (30–45 min)	Purpose: In this phase, we aim to delve deeper into the impact of responding to an OHCA alert and how this may change due to the smartwatch technology. First, we will provide feedback from the previous round through a presentation that summarises the previously mentioned advantages and disadvantages for community responders regarding the technology. The goal is to provide more context for the upcoming discussion. A set of relevant questions will be shared, which the participants could pick to elaborate on. Experiences will be shared, and a connexion will be made to smartwatch technology, focusing on how it should be designed to best support community responders.
Closing (0–5 min)	“With this, we have come to the end of this session. I would like to thank you very much for your effort and time. We are very pleased with your contribution to the development process of smartwatch technology for cardiac arrest detection. We would like to emphasise once again that we will handle the data confidentially. If there are any questions, you can ask them now.” “We are also very curious about your opinion on this session. What did you find positive? And what could be improved in the future?” “Once again, thank you very much for coming and participating in the discussion. We wish you a pleasant day and a safe journey home.”

**Figure:**
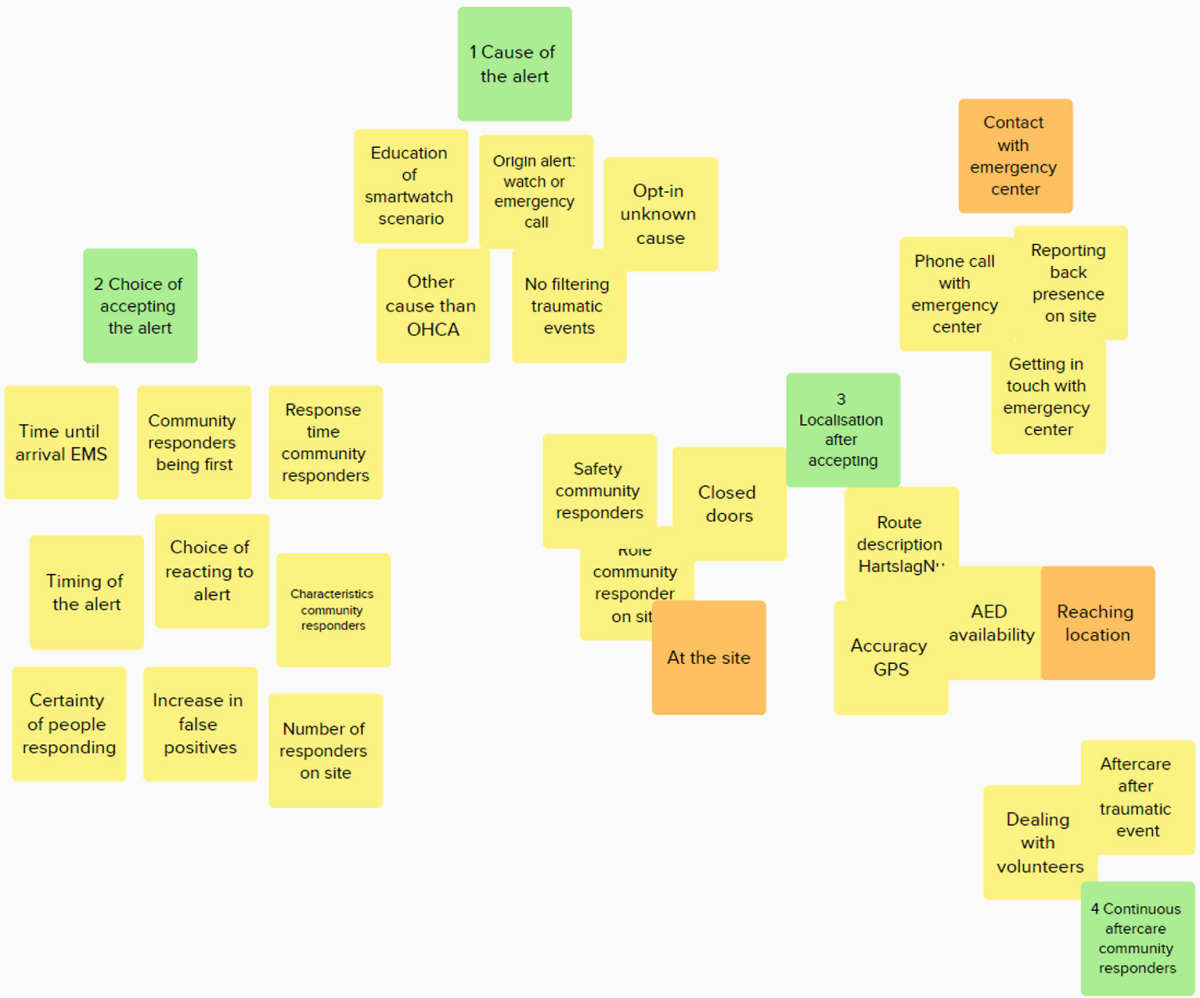


## Appendix A Focus group protocol

Research question: What is the perspective of community responders on smartwatch technology aimed at detecting out-of-hospital cardiac arrest (OHCA)?

Sub-question 1: How does the use of smartwatches for OHCA detection fit into the current resuscitation infrastructure?

Sub-question 2: What is the current impact of responding to a resuscitation emergency call? And how could this change as a result of implementing smartwatch-based OHCA detection?
